# Follow-up Study of 17-β Estradiol, Prolactin and Progesterone with the Kinetics and Prevalence of *T. gondii* Infection in Pregnant Women

**DOI:** 10.3390/cimb46060341

**Published:** 2024-06-07

**Authors:** Yithzel Guadalupe Luna Rojas, Eva Elizabet Camarena Pulido, Laura Rocío Rodríguez-Pérez, María de la Luz Galván-Ramírez

**Affiliations:** 1 Departamento de Ginecología y Obstetricia, Hospital Civil Juan I. Menchaca, Guadalajara 44340, Mexico; 2 Departamento de Microbiología y Patología, Centro Universitario de Ciencias de la Salud, Universidad de Guadalajara, Guadalajara 44340, Mexico

**Keywords:** toxoplasmosis, intrauterine growth restriction, 17-β estradiol, progesterone, prolactin

## Abstract

Toxoplasmosis is an infection caused by the parasite *Toxoplasma gondii.* One-third of the world’s population has come into contact with this parasite. In Mexico, the prevalence is between 15% and 50% in the general population and 34.9% in women with high-risk pregnancies. In pregnancy, the highest incidence of infection occurs in the third trimester and fetal damage is inversely proportional to gestational age. Maternal hormones play a fundamental role in the immune response. There are very few studies, with controversial results, on the levels of increased hormones and their relationship to the kinetics of *T. gondii* infections during pregnancy. The aim was to determine the serum levels of 17-β estradiol, prolactin, and progesterone, and their association with anti-*T. gondii* antibodies’ kinetics in pregnancy. Fifty-two pregnant patients were studied. A questionnaire with sociodemographic and clinical aspects was used. Afterward, 10 mL of venous blood was collected by venipuncture every trimester. The concentrations of 17-β estradiol, progesterone, and prolactin were measured, using the ELISA method. In addition, anti-*Toxoplasma* IgG and IgM antibodies were also determined in the first, second, and third trimester. The prevalence of anti-*Toxoplasma* IgG antibodies was 26.92% in the first and second trimester and 32.7% in the third trimester. In seropositive women, 17-β estradiol increased in the second and third trimesters of pregnancy. Progesterone increased significantly *p* < 0.039 in the third trimester in these women, while prolactin increased in the second trimester with a statistical significance of *p* < 0.021. In addition, 17-β estradiol, progesterone, and prolactin are associated with *T. gondii* infection during pregnancy. New studies are necessary to clarify the specific mechanisms of immune response related to these hormones during pregnancy.

## 1. Introduction

Toxoplasmosis is a disease caused by the protozoan *Toxoplasma gondii* (*T. gondii*). The worldwide prevalence depends on geographic location, dietary habits, and cohabitation with cats [1]. The infection can be asymptomatic. The worldwide prevalence in women varies between 7% and 51%. In women who have had at least one abortion, the prevalence is 17–52.3% [2]. In Mexico, a meta-analysis was carried out in 2012 in which the average prevalence was 27.97% and the weighted prevalence was 19.27%. The latter was higher in women with a history of miscarriage (35.13%), immunocompromised patients (28.54%), and psychiatric patients (38.52%) [3].

Congenital toxoplasmosis occurs when a woman acquires the infection for the first time in a period shortly before or during pregnancy [1,2,3]. In the acute phase, the tachyzoites of *T. gondii* penetrate the placental barrier and infect the fetus. Nonetheless, maternal immunity exists if contact with the parasite has already taken place before pregnancy.

The incidence of primary infection in pregnancy varies between 1 in 310 to 1 and 10,000 pregnancies. The probability of transplacental infection and the clinical manifestations depend on the trimester of pregnancy in which the mother becomes infected, and the severity of damage to the fetus is inversely proportional to the gestational age at which the infection occurs [4,5,6,7,8,9,10].

Regarding 17-β estradiol (E2), pregnancy is considered as a state of immunological tolerance. Several mechanisms have been found to contribute to this tolerance [11,12]. There is evidence of progressive maternal immunoregulation at the interface, which affects the decidua and placentation. Some specific gestational factors, such as maternal hormones, can modulate the maternal immune response and influence the activation of macrophages and lymphocytes in the decidua [13]. There is a marked increase in E2 and progesterone (P4) in the second and third trimesters of pregnancy, and it is during this period that the prevalence of *Toxoplasma* infection is the highest [14]. Endocrine changes during pregnancy, as well as the size and development of the placenta, affect the mother’s ability to fight infections [15]. E2 is mainly synthesized in the ovaries, breast tissue, endometrium, and brain. E2 plays an important role in the menstrual cycle and human reproduction. On the other hand, increased levels of E2 have also been associated with toxoplasmosis during pregnancy [16,17,18,19].

Progesterone (P4) is synthesized in the ovaries and the corpus luteum. It is mainly involved in the second phase of the menstrual cycle and is vital in the first weeks of pregnancy [20]. Progesterone plays an important role in regulating the immune cells, which are essential for pregnancy maintenance. The development of the decidua also depends on progesterone, being crucial for the trophoblast implantation [21,22]. A decrease in serum P4 levels has been observed in pregnant women infected with *T. gondii*; however, the mechanism by which this modulation occurs is still unknown [22,23,24].

Prolactin (Prl) is secreted by the pituitary gland, but it is also produced by the placenta, B and T lymphocytes, and NK cells. Its secretion is controlled by the PrL Inhibiting Hormone (PIH), which is dopamine. Both men and women have low serum levels of this hormone [23,24]. Hyperprolactinemia is a situation in which high levels of Prl are present in the blood, which is very common in women, especially during pregnancy [25,26]. The differences observed in the prevalence of some parasitic infections between men and women may indicate the possible role of sex hormones in the immunity against these parasites. One of the hormones that exhibit a broad spectrum of biological activities, including immunomodulatory effects, is Prl [25,26,27]. In addition, it has been documented, # that female patients with hyperprolactinemia have a lower prevalence rate of *T. gondii* infections compared to those with hipoprolactinemia [27].

The aim was to determine the serum levels of 17-β estradiol, prolactin, and progesterone, and their association with anti-*T. gondii* antibodies kinetics in pregnancy.

## 2. Materials and Methods

### 2.1. Study Design

It was an observational and longitudinal clinical study in pregnant women. The target population were patients attending prenatal obstetric appointments at the Nuevo Hospital Civil de Guadalajara “Dr. Juan I. Menchaca” from 2021 to 2023. The patients were invited to participate and were informed about the study. Patients who agreed signed an informed consent form. 

### 2.2. Inclusion Criteria

A total of sixty pregnant women, with a range of age of 17 to 42 years, participated in this study. The women were informed about all aspects of the study. Those who agreed to participate signed their informed consent. A questionnaire was then completed, which included answers on the risk factors for *T. gondii* infection. In addition, 10 mL of venous blood were collected at weeks 5–13.6 (first trimester), 14–25.6 (second trimester), and 26–40 (third trimester) of pregnancy. 

### 2.3. Exclusion Criteria

Women who decided not to participate were excluded. In addition, women with an obstetric emergency, e.g., severe pre-eclampsia, eclampsia, placental abruption, uterine rupture, inevitable pregnancy loss, ectopic pregnancy, and multiple pregnancies were also excluded. 

### 2.4. Elimination Criteria

Eight patients that stopped showing up to the follow-up appointments and did not show up for the next blood draw were excluded. 

### 2.5. Questionnaire

Occupation (household, professional, employee, student, and merchant). Education (none, elementary school, middle school, high school, bachelor’s degree, master’s degree). Living situation (rural or urban). Features of the house (dirt floor, mosaic floor, electricity, water, and drainage). Contact with cats in the house (no, yes, indoors or outdoors, use of a litter box). Handling cat feces (with gloves, without gloves). Handling food (washed or unwashed vegetables and fruit, raw or cooked vegetables). Background information (blood transfusions, miscarriage or stillborn) Clinical features (visual disturbances, headaches, and lymphadenopathy). 

### 2.6. Diagnostic Laboratorio

Blood Samples: The blood that was drawn was centrifuged to obtain the serum and stored at a temperature of −20 °C until processed. 

#### 2.6.1. Immunoenzyme Assay (ELISA) for Anti-Toxoplasma IgG and IgM

ELISA kits were used (Platelia TM Toxo; Bio-Rad, IgG catalog # 72840 and IgM 72841, Marnes-la-Coquette, France). The standard control and samples were diluted 1/21. The microplate is sensitized with the inactivated T. gondii antigen. IgG and IgM antibodies against Toxoplasma were determined in the first, second, and third trimester of pregnancy. The antibody concentrations were determined according to the manufacturer’s instructions. The coefficient of variation intra-assay and inter-assay for IgG was 5.96% and 10.2% respectively, the sensitivity of IgG was 98.3%, and the specificity was 100%. Titer ≥ 9 IU/mL was considered positive. Regarding the IgM antibodies, only 1/60 samples (1.96%) were positive. Concerning IgM, the intra-assay and the inter-assay coefficient of variation were 5.96% and 10.1% respectively. The sensitivity was 93% and the specificity was 99.9%.

#### 2.6.2. Immunoenzyme Assay (ELISA) for Serum Levels of 17β-Estradiol, Prolactin and Progesterone

The serum levels of E2, Prl, and P4 were determined by a Monoclonal immunoenzyme assay (ELISA Monobind, Inc, Lake Forest, CA, USA, E2 4925-300, PRL 725-300, and PG 4825-300). All samples were analyzed in duplicate. The three assay tests had the same principle. Samples, standards, and controls were duplicated and analyzed according to the manufacturer’s instructions. The samples analyzed were from the first, second, and third trimesters of pregnancy. 17β-estradiol had a sensitivity of 95% and a specificity of 98%. The inter-assay coefficient of variation was 8.6%. For progesterone, the sensitivity was 95% and the specificity 96%. The Inter-assay coefficient of variation was 6.2%. And for Prl the sensitivity was 95% and the specificity 96%. The inter-assay coefficient of variation was 5.2%. Titer ≥ 1.00 were considered positive. 

### 2.7. Statistical Analysis

Data analysis was performed using SPSS software version 27 (IBM, Los Angeles, CA, USA). Variables such as IgG anti-Toxoplasma antibodies between positive and negative pregnant women were analyzed using Pearson’s chi-square test or Fisher’s exact probability using an ANOVA test. The risk of infection due to Toxoplasma exposure was determined using odds ratio tests with a 95% confidence interval to assess the risk, and the significance level was *p* < 0.05.

## 3. Results

### 3.1. Risk Factors and Sociodemographic Features

A questionnaire with sociodemographic data, risk factors, and comorbidities was answered by 52 patients, who attended prenatal appointments in the first, second, and third trimesters. Therefore, the results of anti-*Toxoplasma* IgG, E2, P4, and Prl concentrations were obtained per trimester.

The association of anti-*Toxoplasma* antibodies was examined with occupation (household, professional, employee, student, and merchant); education (none, elementary school, middle school, high school, bachelor’s degree, master’s degree); living situation (rural or urban); features of the house (dirt floor, mosaic floor, electricity, water, and drainage); contact with cats in the house (no, yes, indoors or outdoors, use of a litter box); handling cat feces (with gloves, without gloves); and handling food (washed or unwashed vegetables and fruit, raw or cooked vegetables). No statistically significant difference was found in the socio-demographic data between seropositive and seronegative patients. See Supplementary Table S1.

#### Clinical Features

The mean age was 24.9 ± 5.61 years, with a range of 17 to 35 years, and 15/52 (28.84%) of patients had a prior history of miscarriage or stillborn, of which 33.3% were positive for anti-*T. gondii* IgG. The risk of having a miscarriage or stillborn was 1.8 times higher in patients seropositive to IgG (OR: 1.16, CI 95%: 0.28–4.48). Background information included clinical features (visual disturbances, headaches, and lymphadenopathy, miscarriage or stillborn). No significant risk was found related to seropositivity and visual disturbances, headaches, or lymphadenopathy.

### 3.2. Prevalence of Anti-Toxoplasma Antibodies

**Figure 1:**
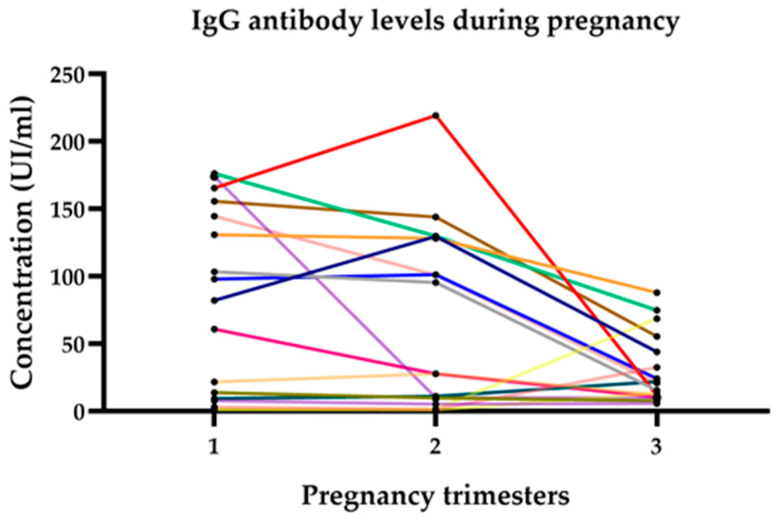
The kinetics of anti-*Toxoplasma* IgG antibodies, with high levels in the first trimester and a tendency to decrease in the second and third trimesters of pregnancy. Concentration greater than 200 IU/mL corresponds to a patient with acute infection who was also positive for IgM (red line).

By pregnancy trimester, seropositivity for anti-*Toxoplasma* IgG was 14/52 (26.92%) in the first and second trimesters and 17/52 (32.7%) in the third trimester. Concerning IgM, only 1/52 (1.92%) were positive in the first trimester. High levels of anti-*Toxoplasma* antibodies were observed in the first trimester. The majority increased more than four times compared to the cut-off value, and a decrease during the second and third trimesters was observed in Figure 1.

The comparison of the serum levels of E2, Prl, and P4 in patients seropositive to anti-*Toxoplasma* IgG antibodies, against those that were seronegative, was performed in each trimester of pregnancy and showed the following significant result: an important rise in Prl levels in the second trimester with a *p* value < 0.021, and increase in P4 levels in the third trimester with a *p* value < 0.039. The colored lines correspond to each of the seropositive patients.

### 3.3. Hormonal Levels

The concentration of IgG and IgM antibodies was reported in IU/mL according to the manufacturer’s instructions. Similarly, E2 concentration values were expressed in pg/mL, while those of Prl and P4 were expressed in ng/mL. The analysis of the ranges and mean standard deviation of the anti-*Toxoplasma* antibodies and hormones can be found in Table 1.

**Figure 2:**
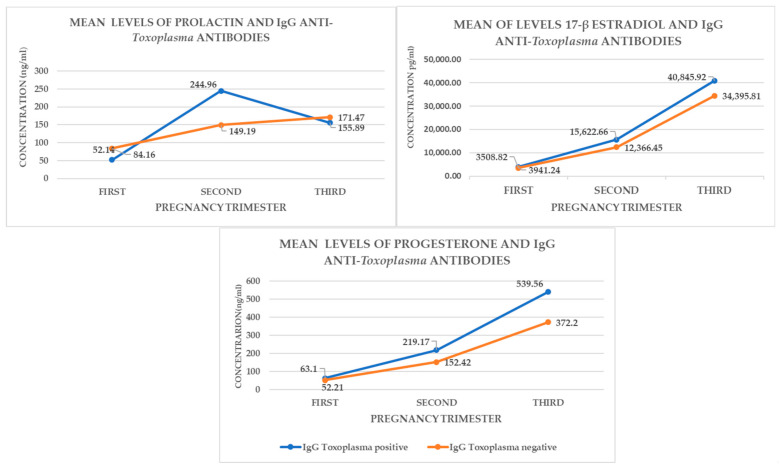
Mean of levels of anti-*Toxoplasma* IgG antibodies, associated with hormone mean levels. The mean of P4 in seropositive women was higher than negatives *p* < 0.039 by pregnancy trimester.

To determine the hormonal behavior of E2, Prl, and P4, related to the positivity of anti-*T*. *gondii* IgG antibodies, by trimester of pregnancy, an ANOVA test was calculated (Figure 2).

**Figure 3:**
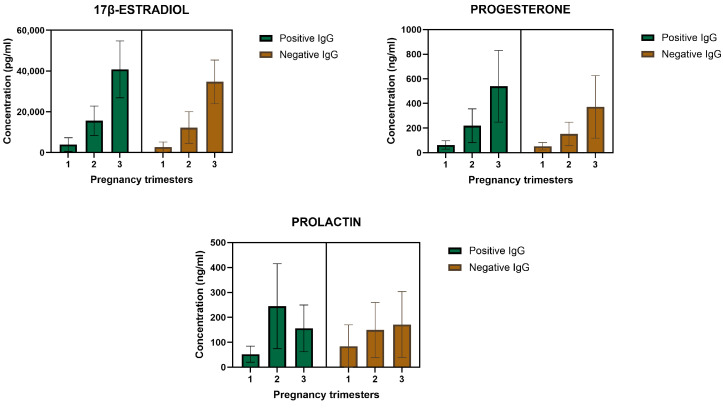
Hormone concentration and association with positive and negative antibodies for anti-Toxoplasma. Progesterone had a sustained increase per trimester of gestation *p* < 0.39 while prolactin decreased in the third trimester *p* > 0.021.

In the second trimester, Prl showed a significant increase (*p* < 0.02), while P4 was also raised, but without reaching statistical significance, with a *p* value of 0.053. On the other hand, P4 showed a significant increase (*p* < 0.039) in the third trimester of pregnancy (Table 2, Table 3 and Table 4). The concentration of E2, P4, and Prl and association with positivity of anti-*Toxoplasma* antibodies are shown in Figure 2 and Figure 3 and Table 2, Table 3 and Table 4.

**Figure 4:**
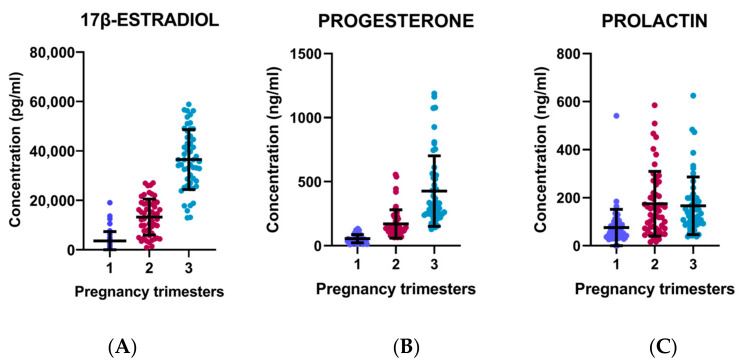
The increase in hormone concentrations in all patients, with and without *T. gondii* infection (**A**) 17β-estradiol, (**B**) progesterone, and (**C**) prolactin, per trimester of gestation.First trimester (Purple Color), second trimester (Wine Color) and Third trimester (Blue Color).

E2 levels in all patients show a gradual increase of this hormone per trimester, being slightly more pronounced in the third trimester. In addition, P4 levels increased significantly in the second and third trimesters, while Prl increased similarly, only more pronounced, in the second trimester (Figure 4).

## 4. Discussion

The prevalence of *T. gondii* infection in the population studied was 26.9% in the first and second trimesters, rising to 32.7% in the third trimester. This was lower than the 34.9% found in women with high-risk pregnancies in 1995 and close to the 26.01% found in the control group of the same population. On the other hand, only one case of IgM seropositivity was found (1.92%); lower than the 20.7% found in 350 women with high-risk pregnancies and close to the 1.9% in the control group of this study [8]. This could be due to the increased awareness, over the last 30 years, of the risks of neonatal infection during pregnancy. However, the incidence found is higher than that reported in another study from Venezuela, where IgM was positive in 0.26–0.47% of the studied population [2].

Regarding hormonal E2 levels, a trend towards higher E2 levels was observed in women seropositive to IgG, without reaching statistical significance. This finding is similar to that of pregnant women from Iraq and Egypt, where higher E2 levels were associated with chronic *T. gondii* infection [20,22]. This suggests that E2 increases maternal susceptibility to *T. gondii* infections [19].

As for acute infection, only one case was IgM-positive, so we could not compare our results between IgG seropositive and acute infections and could not predict whether E2 levels were related to the kinetics of the acute illness. In studies conducted in vitro, pretreatment with E2 on THP-1 cells infected with *T. gondii* increased parasite replication [16,17]. In addition, another study found that E2 can promote the infection by *T. gondii* in vitro and in vivo, which is related to the *Toxoplasma* gene Tg-HSD [18]. On the other hand, E2 can decrease the inflammation [28,29,30].

The P4 levels were higher in the seropositive IgG patients, compared to the seronegative patients, in the second and third trimesters of pregnancy. These findings are similar to those reported in Iraq in pregnant women infected with *T. gondii*, where an increase in P4 levels was found in the second and third trimesters compared to controls [21]. Furthermore, low P4 levels in pregnant patients with toxoplasmosis are associated with adverse effects caused by the infection, as was demonstrated [13]. On the other hand, it has been suggested that the *T. gondii* membrane receptor protein (TgPGRMC), located in the parasite’s mitochondria, may be affected by P4, inhibiting its replication [24].

These results are consistent with those found in a study executed in vivo, in which P4 levels were strongly associated with chronic infection [21]. In another study carried out on in vitro astrocytes that were pretreated with P4 and infected by *T. gondii*, it was found that the P4 treatment reduced the parasitic replication significantly, compared to the control group [15].

As for the results of Prl levels, they increased in the second and third trimesters of pregnancy in the overall population. However, Prl levels were significantly higher in IgG-positive women in the second trimester, while an important decrease is observed, in this group, in the third trimester. Similar results were reported in a study carried out on pregnant women with *T. gondii* infection in which they observed a drop in Prl levels in the second and third trimesters [22]. In contrast, a study conducted on non-pregnant women in Iran found a lower prevalence of *T. gondii* infections in patients with high Prl levels [25]. The same was observed in a study from India, in which they also found a lower prevalence of IgG seropositivity in non-pregnant women with high Prl levels [27]. On the other hand, a study showed an inhibitory effect of Prl on the replication of *Toxoplasma* in peripheral blood mononuclear cells of patients with hyperprolactinemia [26].

Furthermore, in a study executed on THP-1 cells pretreated with Prl (200 ng/mL), E2 (40 nM), and P4 (40 nM) and infected with *T. gondii* tachyzoites, in which the hormonal receptor expression (PrlR, ERα, and ERβ) and 17 cytokines were evaluated, it was observed that Prl did not alter the production of IL-12 or IL-1β, but increased IL-10, IL-4 and IL-13 and decreased the expression of ERα, Erβ, demonstrating the importance of Prl in the immune response against a *Toxoplasma* infection [16,17].

Previous studies suggest that Prl plays a fundamental role in the immune response against *T. gondii* infection. However, the decrease in Prl levels in seropositive patients in the third trimester of pregnancy, which was found in this study, is not completely clear and may be due to the modulation of stress responses during pregnancy.

With regards to the clinical features, 31.6% of patients had a history of miscarriage or stillborn, of which 36.8% were seropositive for anti-*Toxoplasma* IgG, lower than the 44.9% found in women with repeated miscarriage in 1995 [1,8].

Finally, E2 can play a dual role in *T. gondii* infection. It promotes infection by activating the parasite’s Tg-HSD gene and decreases inflammatory processes through the ERα receptor. Recombinant prolactin rhPRL could play a protective role by inhibiting parasite replication and modulating cytokine production TNF-α, IFNγ, and IL-12, which has been previously demonstrated. Progesterone has a protective and immunomodulatory role during pregnancy, protecting the fetus. The progesterone receptor (PGRMC) can regulate and promote the growth, proliferation, and differentiation of *Toxoplasma*. The *T. gondii* progesterone receptor membrane protein (TgPGRMC) can interact with E2 receptors ERα and ERβ. Progesterone can reduce parasite replication through GPER, decreasing the secretion of the parasite’s MIC2 protein while reducing its pathogenicity.

Future studies are necessary to understand other molecules and their mechanisms and signaling pathways that allow us to know the behavior of estradiol, prolactin, and progesterone in Toxoplasma infection during pregnancy.

## 5. Conclusions

E2 levels increased, without a relationship with the presence of anti-*T. gondii* antibodies, during pregnancy.

Progesterone is associated with high levels of IgG anti-*Toxoplasma* antibodies in the third trimester of gestation.

Prolactin levels in the second trimester decreased in seronegative women.

The hormones 17β-estradiol, progesterone, and prolactin may be immunomodulators in *T. gondii* infection during pregnancy.

## Figures and Tables

**Table 1:** Quarterly anti-*Toxoplasma* antibodies and hormone mean.

	*N*	Minimum	Maximum	Mean	Standard Deviation
1T *Toxoplasma* IgG UI/mL	52	0.86830	176.35000	27.11175	53.25078
2T *Toxoplasma* IgG UI/mL	52	0.83000	219.33000	23.05660	49.10734
3T *Toxoplasma* IgG UI/mL	52	0.83000	111.67000	12.96396	24.42573
1T *Toxoplasma* IgM UI/mL	52	0.04477	1.43461	0.16810	0.21263
2T *Toxoplasma* IgM UI/mL	52	0.01684	0.46863	0.14868	0.09057
3T *Toxoplasma* IgM UI/mL	52	0.05472	0.48979	0.14377	0.09230
ESTRADIOL 1T pg/mL	52	376.72512	19,033.90000	3625.24431	3722.97799
ESTRADIOL 2T pg/mL	52	938.72444	27,051.50000	13,243.12995	7285.86553
ESTRADIOL 3T pg/mL	52	12,914.73	58,901.15	36,504.5008	12,114.67400
PROLACTIN 1T ng/mL	52	0.53000	540.98700	75.54135	76.42632
PROLACTIN 2T ng/mL	52	15.38000	584.96000	174.98144	134.67540
PROLACTIN 3T ng/mL	52	38.46000	625.00000	166.37818	120.15226
PROGESTERONE 1T ng/mL	52	10.791366	133.20000	55.14779	31.47740
PROGESTERONE 2T ng/mL	52	65.10791	555.29000	170.394048	110.82653
PROGESTERONE 3T ng/mL	52	129.439252	1187.83783	426.91970	275.42972

**Table 2:** Hormonal levels associated with anti-*Toxoplasma* IgG antibodies during the first trimester of pregnancy.

Hormone TOXO	IgG	*N*	Mean	Standard Deviation	Standard Error	CI 95% Media		*p*
						Lower Bound	Upper Bound	
ESTRADIOL	positive	14	3941.24243	3326.45899	889.03356	2020.602	5861.882	
	negative	38	3508.82395	3894.30121	631.73907	2228.798	4788.848	0.714
	Total	52	3625.24431	3722.97799	516.28415	2588.760	4661.728	
PROLACTIN	positive	14	52.147164	32.48556	8.68213	33.390	70.903	
	negativo	38	84.16026	86.00537	13.95191	55.891	112.429	0.183
	Total	52	75.54135	76.42632	10.59842	54.264	96.818	
PROGESTERONE	positive	14	63.10866	33.85084	9.04701	43.563	82.653	
	negative	38	52.214846	30.50103	4.94792	42.189	62.240	0.273
	Total	52	55.14779	31.47740	4.36513	46.384	63.911	

**Table 3:** Hormonal levels associated with anti-*Toxoplasma* IgG antibodies during the second trimester of pregnancy.

Hormone TOXO	IgG	*N*	Mean	Standard Deviation	Standard Error	CI 95% Media		*p*
						Lower Bound	Upper Bound	
ESTRADIOL	positive	14	15,622.66740	7179.86307	1918.89912	11,477.13788	19,768.19691	
	negative	38	12,366.45826	7219.80052	1171.20630	9993.36888	14,739.54764	0.155
	Total	52	13,243.12995	7285.86553	1010.36776	11,214.73203	15,271.52787	
PROLACTIN	positive	14	244.96568	170.40965	45.54389	146.57408	343.35728	
	negative	38	149.19778	110.730559	17.96286	112.80155	185.59401	**0.021**
	Total	52	174.98144	134.67540	18.67611	137.48757	212.47532	
PROGESTERONE	positive	14	219.17293	137.32029	36.70039	139.88656	298.45931	
	negative	38	152.42287	95.25009	15.45160	121.11495	183.73080	0.053
	Total	52	170.39404	110.82653	15.36887	139.53974	201.24835	

**Table 4:** Hormonal levels associated with anti-*Toxoplasma* IgG antibodies during the third trimester of pregnancy.

Hormone TOXO	IgG	*N*	Mean	Standard Deviation	Standard Error	CI 95% Media		*p*
						Lower Bound	Upper Bound	
ESTRADIOL	positive	17	40,845.9224	13,959.58813	3385.69743	33,668.5644	48,023.2803	
	negative	35	34,395.8103	10,697.80746	1808.25950	30,720.9848	38,070.6357	0.071
	Total	52	36,504.5008	12,114.67400	1680.00301	33,131.7540	39,877.2475	
PROLACTIN	positive	17	155.89054	93.587113	22.69820	107.77249	204.00859	
	negative	35	171.47218	132.10402	22.329655	126.092862	216.85150	0.665
	Total	52	166.378184	120.152262	16.66212	132.92758	199.828787839996270	
PROGESTERONE	positive	17	539.56442	290.48655	70.45333	390.21001	688.91882	
	negative	35	372.20655	254.27726	42.98070	284.85926	459.55385	**0.039**
	Total	52	426.91970	275.42972	38.195230	350.23958	503.59983	

## Data Availability

Data are contained within the article.

## Supplementary Materials

The following supporting information can be downloaded at: https://www.mdpi.com/article/10.3390/cimb46060341/s1, Table S1: Sociodemographic variables and risk factors with anti-*toxoplasma* IgG antibodies.
