# Supplementary material for: Follow-up Study of 17-β Estradiol, Prolactin and Progesterone with the Kinetics and Prevalence of T. gondii Infection in Pregnant Women
Source: Curr Issues Mol Biol. 2024 Jun 7;46(6):5701–11. doi: 10.3390/cimb46060341 (PMC11202031; doi:10.3390/cimb46060341)
Supplement: Supplementary file 1 [file cimb-46-00341-s001.zip › cimb-3020158-supplementary.pdf]

TABLE S1. SOCIODEMOGRAPHIC VARIABLES AND RISK FACTORS WITH ANTI-*Toxoplasma* IgG ANTIBODIES

VARIABLE	IGG TOXOPLASMOSIS POSITIVO.	%	NEGATIVO.	%	X^2	P	OR	IC 95%
OCUPACIÓN								
HOME	8	28.6	20	71.4				
PROFESSIONALIST	1	25.0	3	75				
EMPLOYEE	1	25	3	75	3.47	0.628		
STUDENT	2	16.7	10	83.3				
BUSINESSMAN	2	66.7	1	33.3				
TOTAL	14	26.9	38	73.1				
SCHOLARSHIP								
NONE	1	1.9	0	0				
PRIMARY EDUCATION	1	33.3	2	66.7				
SECONDARY	6	35.3	11	64.7	4.51	0.478		
BACHELOR	4	21.1	15	78.9				
DEGREE	2	18.2	8	81.8				
POSGRADUATE	0	0	1	100				
TOTAL	14	26.9	38	73.1				
HOUSING								
RURAL	1	50	1	50				0.55-
URBAN	13	26.0	37	74.0	0.453	0.489	2.625	44.52
TOTAL	14	26.9	38	73.1				
FLOOR								
LAND	0	0	1	100				
MOSAIC	8	25.0	24	68.4	0.638	0.727		
CEMENT	6	31.6	13	63.3				
TOTAL	14	26.9	38	73.1				
LIGHT								
YES	14	26.9	38	73.1	CONSTANTE			
NO	0	0	0	0				
TOTAL	14	26.9	38	73.1				
WATER								
YES	12	24.5	37	75.2				
NO	2	66.7	1	33.3	2.285	0.131	0.179	0.015-
TOTAL	14	26.9	38	52				2.115
DRAINAGE								
YES	13	26.5	36	73.5				
NO	1	33.3	2	66.7	0.066	0.618	0.78	0.66-
TOTAL	14	26.9	38	73.1				9.218
CATS								
YES	4	40.0	6	60.0				
NO	10	23.8	32	76.2	0.428	0.254	2.143	0.572-
TOTAL	14	26.9	38	73.1				8.026
CATS IN HOME								
NO	10	23.8	32	76.2				
YES	4	40.0	6	60.0	2.246	0.325		
TOTAL	14	26.9	38	73.1				
USE SAND BOX								
NO CATS	12	25	36	75				
YES	1	25	3	75	1.939	0.399		
NO	3	50.0	3	57.1				
TOTAL	14	26.9	38	73.1				
USING GLOVES TO CLEAN FECES								
NO CATS								
YES	10	23.8	32	76.2				
NO	2	50	2	50	1.415	0.493		
TOTAL	2	33.3	4	66.7				
	14	26.9	38	73.1				
CONSUMPTION OF FRUITS AND								
VEGETABLES								
YES	14	26.9	36	72.0	0.39	0.530	0.78	0.066-
NO	0		2	110.0				9.21
TOTAL	14	26.9	38	73.1				
CONSUMPTION OF UNWASHED								
FRUITS AND VEGETABLES								
YES	1	14.3	6	85.7				
NO	13	28.8	32	71.1	0657	0.383	0.822	0.149-
TOTAL	14	26.9	38	73.1				4.542
TRANSFUSIONS								
YES	1	20.0	4	80.0				
NO	13	27.7	34	72.3	0.447	0.504	0.475	0.51-4.39
TOTAL	14	26.9	38	73.1				
ABORTIONS OR DEATH								
YES	5	33.3	10	66.7				0.559-
NO	9	24.3	28	75.7	0.440	0.368	1.808	5.847
TOTAL	14	26.9	38	73.7				
